# Combined mRNAs and clinical factors model on predicting prognosis in patients with triple-negative breast cancer

**DOI:** 10.1371/journal.pone.0260811

**Published:** 2021-12-29

**Authors:** Yanjun Hu, Dehong Zou

**Affiliations:** Department of Breast Surgery, The Cancer Hospital of the University of Chinese Academy of Sciences (Zhejiang Cancer Hospital), Institute of Basic Medicine and Cancer (IBMC), Chinese Academy of Sciences, Hangzhou, Zhejiang, China; American Society for Investigative Pathology, UNITED STATES

## Abstract

**Objective:**

Triple-negative breast cancer (TNBC) is aggressive cancer usually diagnosed in young women with no effective prognosis prediction model to use. The present study was performed to develop a useful prognostic model for predicting overall survival (OS) for TNBC patients.

**Methods:**

The Cancer Genome Atlas (TCGA) and Molecular Taxonomy of Breast Cancer International Consortium (METABRIC) databases were used as training and validation data sets, respectively, in which the gene expression levels and clinical prognostic information of TNBC were collected. Differentially expressed genes (DEGs) between TNBC and non-TNBC (NTNBC) were identified with the thresholds of false discovery rate < 0.05 and |log_2_ Fold Change| > 1. DEGs in AmiGO2 and the Kyoto Encyclopedia of Genes and Genomes (KEGG) databases were retained for further study. Univariate, multivariate Cox, and logistic regression analysis were conducted for detecting DEG signature with the threshold of log-rank *P* < 0.05. The prognosis models of mRNA signature, clinical factors were constructed and compared.

**Results:**

One five-DEG signature, including *CHST4*, *COCH*, *CST9*, *SOX11*, and *TDGF1* was identified in DEG prognosis model. Stratified analysis showed that the patients aged over 60, with higher pathologic stage (III-IV) and recurrence induced a significantly lower survival rate than those aged below 60, lower pathologic stage and without recurrence. Compared with patients with low-risk scores, those presented high-risk scores demonstrated significantly lower survival rate in the subgroup aged over 60 [HR = 3.780 (1.801–7.933), *P* < 0.0001]. For patients who obtained a higher pathologic stage and recurrence, high-risk scores were correlated with a significantly lower survival rate than patients with low-risk scores. The five-mRNA signature combined with clinical model (AUC = 0.950) predicted better than single clinical model (AUC = 0.795) or five-mRNA signature model (AUC = 0.823).

**Conclusion:**

Our present study identified a prognostic prediction model (combined with five-mRNA signature and clinical factors) for TNBC patients receiving immunotherapy, which will benefit future research and clinical therapies.

## Introduction

Triple-negative breast cancer (TNBC) is an aggressive breast cancer negative for progesterone receptor (PR), estrogen receptor (ER), and human epidermal growth factor receptor 2 (Her-2). As known, TNBC patients benefit little from both endocrine therapy and Her-2 targeted therapy, but chemotherapy, the traditional treatment system [1]. Even worse, TNBC patients suffer from earlier recurrence, worse prognosis, and shorter survival time than other breast cancer subtypes [2–4]. Nowadays, immunotherapy is drawing extensive attention in TNBC tumor therapy [5–8]. The efficacy of immunotherapy in most types of breast cancer has not been confirmed when compared with other cancers with higher immunogenicity as malignant melanoma, pulmonary small cell lung cancer [9, 10]. However, the TNBC immunotherapy approved by FDA in the United States has obtained outstanding curative effects [11]. Although the success of immunotherapy is exciting, countless patients did not respond to immunotherapy.

The complexity and diversity of the tumor microenvironment (TME) had been gradually understood in recent years. Moreover, its importance in immunotherapy has also been realized. TME was the cellular environment, including immune cells, mesenchymal cells, endothelial cells, inflammatory mediators, and extracellular matrix molecules. It is widely considered that the microenvironment plays a significant role in tumor development [12]. Therefore, the comprehensive analysis of the correlation between gene signatures and overall survival (OS) may shed light on the pathogenesis of TNBC.

Chemotherapy remains the most effective therapy method [13]. In recent years, immunotherapy had been widely studied in cancers, especially TNBC. PD-1/PD-L1 are a pair of immune co-stimulatory molecules contain the medicines of pembrolizumab [14], atezolizumab [15], and durvalumab [16], which were reported to be effective for prolonging OS. Clinical studies have found that immune infiltration could improve prognosis in TNBC patients [17, 18]. Therefore, the identification of DEGs of TNBC may contribute to the in-depth analysis of factors affecting survival.

Traditionally, the prognosis was predicted by means of clinical risk factors, including age, tumor size, pathologic stage, and location. With the development of high-throughput sequencing technologies, multigene signatures including miRNA-signature, mRNA-signature, and lncRNA-signature were recognized as valuable biomarkers on predicting breast cancer prognosis, such as Oncotype DX, B-cell/IL8, Mammo-print, and Genomic Grading Index [19–21]. In recent years, an increasing number of studies demonstrated that mRNAs play identification, biomarker, and prognosis prediction roles in TNBC patients [22–27]. However, there was still no mRNA signature associated with prognosis prediction in TNBC patients ever reported.

The Cancer Genome Atlas (TCGA) database and Molecular Taxonomy of Breast Cancer International Consortium (METABRIC) database provide a wealth of available cohorts about cancer-specific gene expression information and detailed clinical characteristics. They accelerated the molecular analysis of cancers. In the present study, the mRNA signature of TNBC was identified by the usage of four databases, including TCGA, METABRIC, AmiGO2, and the Kyoto Encyclopedia of Genes and Genomes (KEGG). Clinical factor and mRNA signature prognosis prediction models were made and compared. The stratified analysis was performed to predict a more accurate prognosis situation. Here, our objective was to find the most accurate and straightforward prognostic model used in the clinical work of TNBC.

In the present study, a five-mRNA signature (*CHST4*, *COCH*, *CST9*, *SOX11*, and *TDGF1*) was constructed to predict the prognosis of TNBC. Moreover, the combined prognosis model of mRNA signature and clinical factors have a better prediction function than a single mRNA signature model or clinical factors.

## Materials and methods

### Clinical information and RNA-Seq dataset in TCGA data set

We downloaded the level three fragments per kilobase million (FPKM) gene-level RNA-Seq data of breast cancer samples produced by Illumina HiSeq 2000 RNA sequencing platform from the TCGA database as a training data set through Genomic Data Commons (GDC) Data Transfer tool (https://portal.gdc.cancer.gov/) before February 01, 2020. The patients with documented complete expression profiles and clinical information, including OS, ER, PR, and Her-2 were selected in the study. This study met the publication guidelines provided by TCGA. At the same time, the gene expression and clinical information of breast cancer samples were downloaded from the METABRIC database (http://molonc.bccrc.ca/) as a validation data set.

### Identification of DEGs

In the TCGA training data set, the differentially expressed genes (DEGs) between TNBC and NTNBC patients were identified by R3.4.1 limma (S1 File) [28] (https://bioconductor.org/packages/release/bioc/html/limma.html) with the thresholds of false discovery rate (FDR) < 0.05 and |log_2_ Fold Change (FC)| > 1. Based on the expression levels of the DEGs, the two-way hierarchical clustering analysis was performed by the centered Pearson correlation algorithm [29] using pheatmap version 1.0.8 (https://cran.r-project.org/web/packages/pheatmap/index.html) [30]. The DEGs in AmiGO2 (http://amigo.geneontology.org/amigo) and KEGG pathway (https://www.kegg.jp/) databases were retained for further study.

### DEGs signature identification and survival prognosis models construction

Univariate and multivariate Cox regression analyses were performed to identify the independent prognosis related DEGs based on the clinical prognosis information (recurrence, dead, OS time) of the samples in the TCGA training data set and gene expression levels of DEGs identified above, using the survival package (version 2.41–1, http://cran.r-project.org/web/packages/survival/index.html) of R3.4.1 (S1 File). Log-rank *P* < 0.05 was considered as the threshold for a significant correlation. Then, the feature DEGs among the above DEGs were screened out using the Logit regression model by glm function in R3.4.1 (S1 File) [31, 32].

The DEG prognostic risk score was calculated based on the expression levels of feature DEGs obtained in the previous steps and the prognostic coefficients of each element in the optimized combination of DEGs. The DEGs prognostic model was calculated as follows:

$$DEGs\;prognostic\;risk\;score=\sum \beta_{DEGs}\times Exp_{DEGs}$$

Here β_*DEGs*_ indicated the coefficient of DEGs derived from multivariate Cox regression, whereas Exp_*DEGs*_ represents the expression level of the target DEGs in the training data set.

Finally, the DEG prognostic risk score of each sample was evaluated. Taking medium value as the threshold, samples were divided into high- and low-risk groups in the TCGA training data set. We compared the real survival prognosis with the grouped samples by DEGs prognostic score model using survival package version 2.41–1 Kaplan–Meier curve method in R3.4.1 (S1 File).

### DEGs prognostic risk model validation analysis

The samples in the TCGA and METABRIC validation data set were both used to validate the performance of this DEG prognostic risk prediction model. The samples in the TCGA training and METABRIC verification data sets were separately classified into TNBC and NTNBC sampling groups by the logistic regression model. The DEGs with *P* < 0.05 were considered as feature DEGs. Then, the accuracy of classification was validated by comparing the predicted group with the actual group.

### Screening for independent prognostic clinical models

The independent prognostic clinical factors in the breast cancer tumor samples of the TCGA training data set were screened using the univariate and multivariate Cox regression analysis in R3.4.1 survival package (version 2.41–1; S1 File). Log-rank *P* < 0.05 was used as the threshold. Then, the stratified analysis was performed.

### Comparison analysis between prognostic clinical factors and DEGs prognostic risk models

To further investigate the correlation of the independent prognostic clinical factors and DEG prognostic risk model, the nomogram analyses of three- and five-year survival rate prediction models were performed. rms package version 5.1–2 of R3.4.1 (S1 File; https://cran.r-project.org/web/packages/rms/index.html) was used [33, 34]. Then, the clinical prognosis models were constructed and compared with the DEGs prognostic model.

## Results

### Data source and preprocessing

Overall, 1,217 samples were assessed, with 710 samples (116 TNBC and 594 NTNBC) with integral clinical information in TCGA training data set were retained for further study. The clinical information statistics are shown in Table 1 and S1 Table. Simultaneously, 299 TNBC and 1,605 NTNBC samples with RNA-Seq expression profiles from the METABRIC database was downloaded as the validation data set.

**Table 1:** The clinical prognosis information statistics of breast cancer samples in the TCGA database.

Variables	TCGA (710)	NTNBC (N = 594)	TNBC (N = 116)	*P*
**Age (years, means ± SD)**	58.52 ± 13.11	59.28 ± 13.27	54.64 ± 11.86	0.00021
**Pathologic_M (M0/M1/-)**	606/9/95	507/7/80	99/2/15	0.647
**Pathologic_N (N0/N1/N2/N3/-)**	338/229/86/49/8	264/203/74/45/8	74/26/12/4/-	0.00266
**Pathologic_T (T1/T2/T3/T4)**	177/420/88/25	151/346/76/21	26/74/12/4	0.760
**Pathologic stage (I/II/III/IV/-)**	118/407/164/9/12	99/334/145/7/9	19/73/19/2/3	0.237
**Radiotherapy (Yes/No/-)**	353/292/65	294/248/52	59/44/13	0.861
**Target-therapy (Yes/No/-)**	320/26/364	264/22/308	56/4/56	0.999
**Recurrence (Yes/No/-)**	46/531/133	33/448/113	13/83/20	0.0375
**Dead (Death/Alive)**	80/630	61/533	19/97	0.0757
**OS time (months, means ± SD)**	36.47 ± 31.18	36.42 ± 31.09	36.76 ± 31.72	0.916

Note: SD: standard deviation; TCGA: The Cancer Genome Atlas; TNBC: Triple-negative breast cancer; NTNBC: non-TNBC; OS, overall survival. M, metastasis status; N, regional lymph node status; T, tumor status.

### Sampling and detection of DEGs

Significant differences were observed in terms of age (*P* = 0.00021), pathologic_N (*P* = 0.00266), and recurrence (*P* = 0.0375) between TNBC and NTNBC patients (Table 1) in TCGA database. No significant differences were detected between TNBC and NTNBC in other clinical factors, including pathologic_M, pathologic_T, pathologic stage, radiotherapy, target-therapy, dead, and OS time (*P* > 0.05; Table 1).

**Fig 1:**
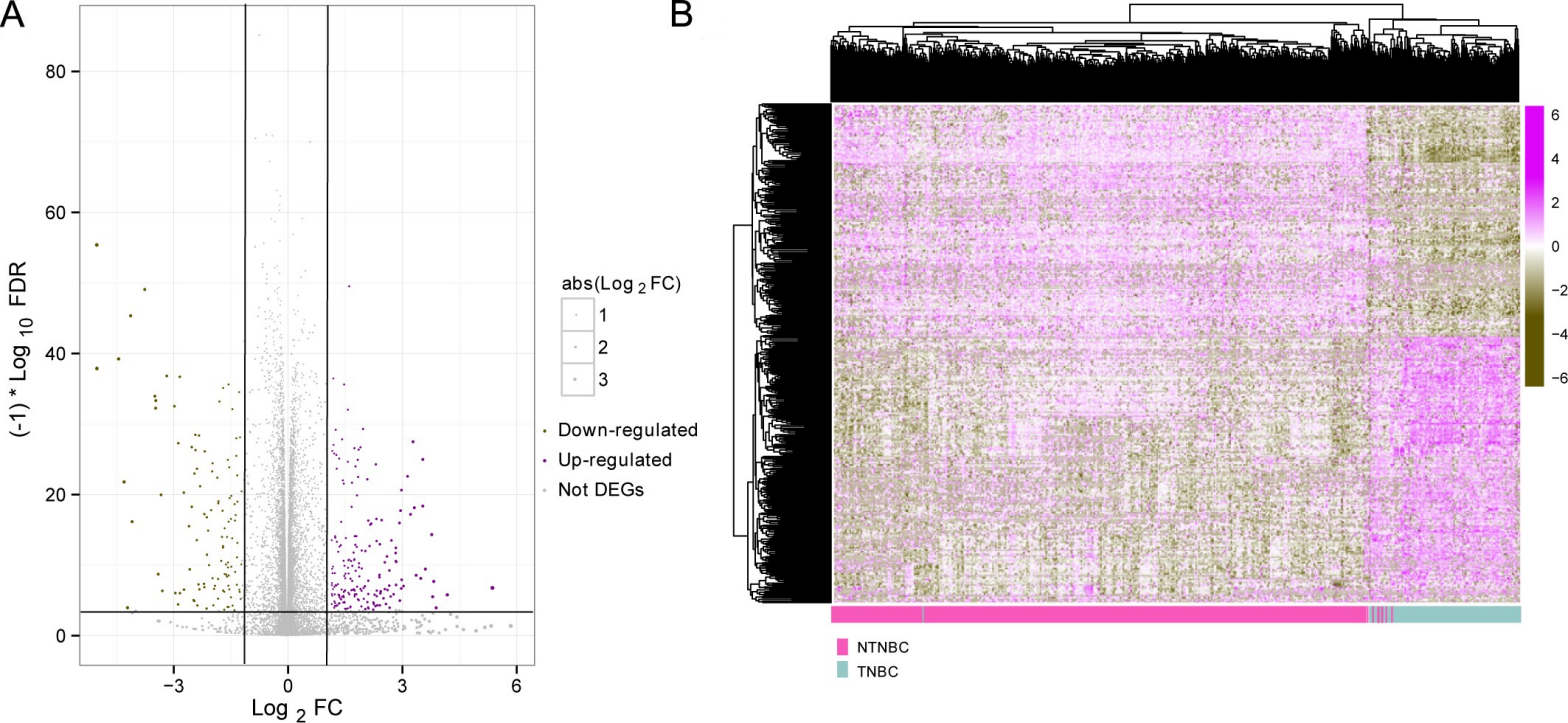
The DEGs detection analysis. (A) The volcano map of the mRNAs. Horizontal dashed lines represent false discovery rate <0.05, and two vertical dashed lines represent |log_2_Fold Change|> 1. The size of the dots represents the absolute log_2_Fold Change, and the larger the value, the larger the point. (B) A two-way hierarchical clustering heat map based on the expression level of DEGs. DEGs, differentially expressed genes.

A total of 15,583 mRNAs were identified in TCGA training data set by removing those with an expression level of 0. With the thresholds of FDR < 0.05 and |log_2_ FC| > 1, 884 DEGs, with 578 up-regulated and 306 down-regulated were identified (Fig 1A; S2 Table). Moreover, the two-dimensional hierarchical clustering heatmap of these DEGs presented an obvious classification on TNBC and NTNBC patients (Fig 1B). A total of 164 DEGs involved in both AmiGO2 and KEGG databases were used for further study (S3 Table).

### Screening of characteristic DEGs and constructing of survival prognosis models

In the TCGA training data set, twenty prognosis-related DEGs were selected through univariate Cox regression analysis (S4 Table), seven of which were left after multivariate Cox regression analysis, including cochlin (*COCH*), carbohydrate sulfotransferase 4 (*CHST4*), LIM domain only 1 (*LMO1*), cystatin 9 (*CST9*), SRY-box transcription factor 11 (*SOX11*), histatin 3 (*HTN3*), and teratocarcinoma-derived growth factor 1 (*TDGF1*) (S5 Table). Thereafter, a five-DEG signature associated with independent prognosis was screened out and used for the Logit regression model construction. These five genes are: *CHST4*, HR = 0.9379 (0.8816–0.9978); *COCH*, HR = 0.8080 (0.7303–0.8939); *CST9*, HR = 0.9499 (0.9127–0.9885); *SOX11*, HR = 1.109 (1.001–1.230); and *TDGF1*, HR = 0.9066 (0.8419–0.9763); Table 2.

**Table 2:** Important DEG signature lists assessed through the Logit regression model and multi-variable Cox regression.

Symbol	Logit regression			Multi-variable Cox regression			
	Estimate	Std. Error	z value	coefficient	HR	lower .95	upper .95
***CHST4***	0.27	0.049	5.51	–0.06409	0.9379	0.8816	0.9978
***COCH***	0.3364	0.0644	5.224	–0.2132	0.8080	0.7303	0.8939
***CST9***	–0.1673	0.02717	–6.158	–0.05143	0.9499	0.9127	0.9885
***SOX11***	0.1872	0.06065	3.087	0.1037	1.109	1.001	1.230
***TDGF1***	0.1245	0.04938	2.521	–0.09805	0.9066	0.8419	0.9763

Notes: DEGs: differentially expressed genes; HR: hazard ratio; *CHST4*: carbohydrate sulfotransferase 4; *COCH*: cochlin; *CST9*: cystatin 9; *SOX11*: SRY-box transcription factor 11; *TDGF1*: teratocarcinoma-derived growth factor 1.

Then, the DEG prognostic risk model was constructed based on the expression profiles of *CHST4*, *COCH*, *CST9*, *SOX11*, and *TDGF1* in the TCGA training data set. The prognostic risk score of each TCGA sample as calculated as: prognostic risk score = (–0.064) × Exp_*CHST4*_ + (–0.213) × Exp_*COCH*_ + (–0.051) × Exp_*CST9*_ + (0.104) × Exp_*SOX11*_+ (–0.098) × Exp_*TDGF1*_.

**Fig 2:**
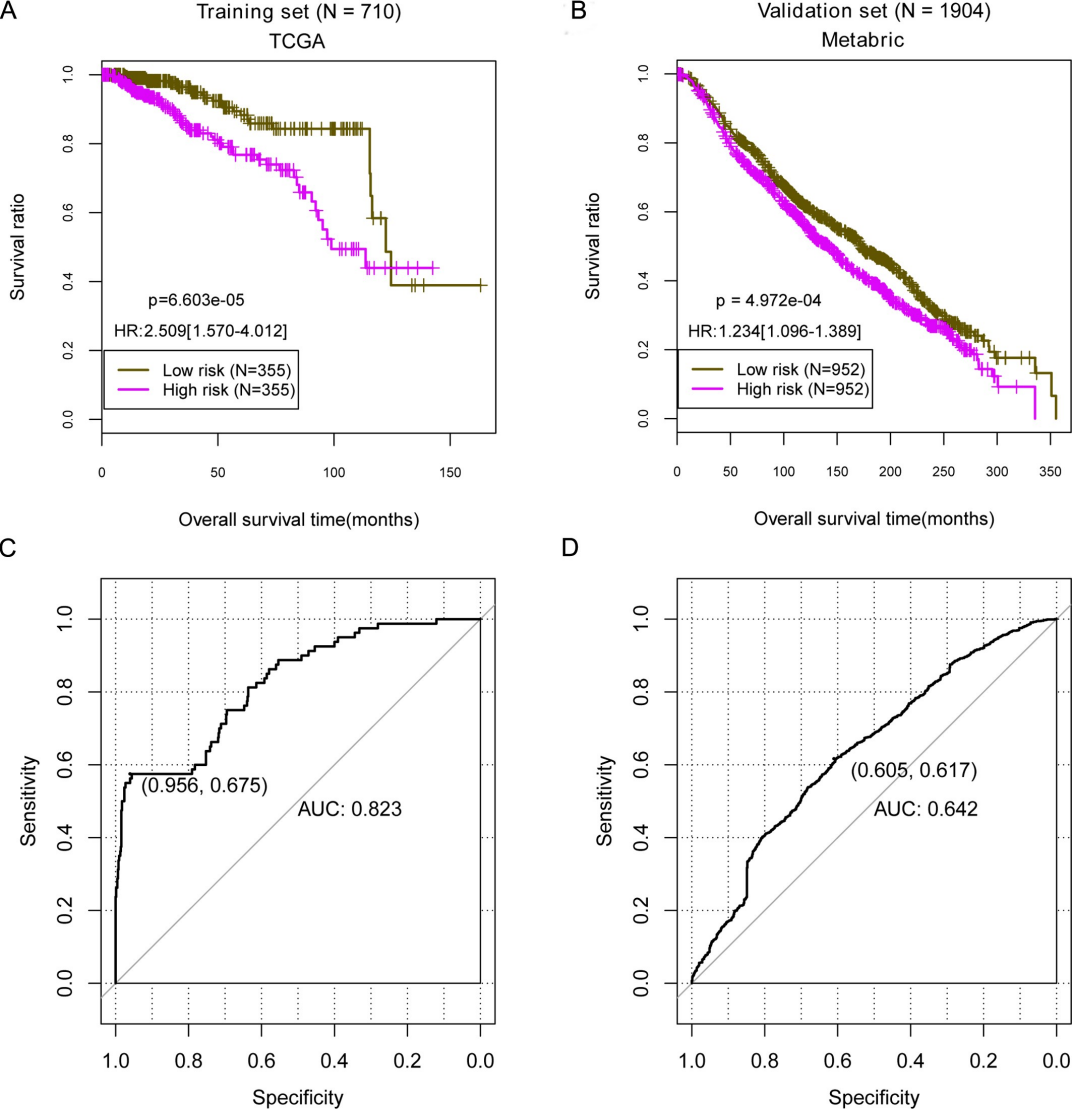
The mRNA prognostic model used the mortality risk score calculation in the TCGA training data set and METABRIC validation set. (A, B) showed the Kaplan-Meier curve based on the mRNA prognostic prediction model and the prognosis in the TCGA training data set and Metabric validation set. Significant differences were observed. (C, D) ROC curve of prediction result based on prognosis model. TCGA, The Cancer Genome Atlas. Metabric, Molecular Taxonomy of Breast Cancer International Consortium. ROC, receiver operating characteristic.

According to the median value of risk scores, samples in each data set were divided into the high- and low-risk groups. Significant differences were observed between high- and low-risk groups in TCGA training data set [HR = 2.509 (1.570–4.012), *P* < 0.0001; Fig 2A]. The patients in the high- and low-risk groups in the METABRIC validation data set also had a difference survival ratio [HR = 1.234 (1.096–1.389), *P* < 0.0001; Fig 2B]. The receiver operating characteristic (ROC) curve was plotted, with the AUC of 0.823 (95% CI: 0.675–0.956; Fig 2C) in the training and 0.642 (95% CI: 0.605–0.617; Fig 2D) in the validation data sets.

### DEGs prognostic risk model validation analysis

**Fig 3:**
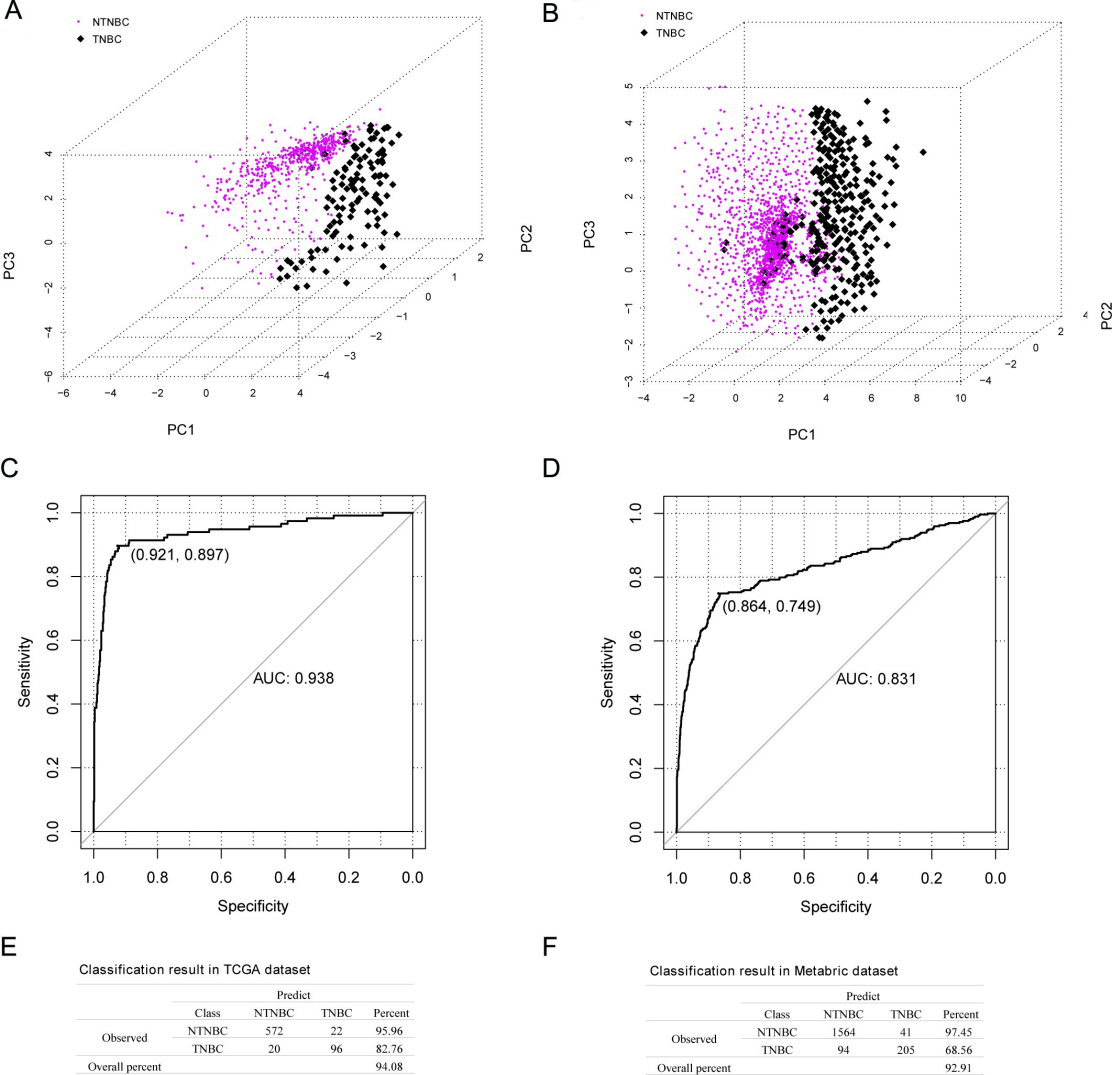
The Logit regression model analysis of TCGA training and METABRIC validation data sets. (A, B) represent the scatter distribution situation of TNBC and NTNBC. (C, D) represent the logistic regression model classification results. Five-mRNA signature precisely identified TNBC and NTNBC samples in the TCGA training and the METABRIC validation data sets. (E, F) exhibited the fuzzy classification matrix result in the TCGA dataset and Metabric dataset, which demonstrated the overall TNBC predictive percent of 94.08% and 92.91% compared to the predicted classification with the actual group. TNBC, triple-negative breast cancer; NTNBC, non-TNBC. TCGA, The Cancer Genome Atlas. Metabric, Molecular Taxonomy of Breast Cancer International Consortium.

The five-DEGs signature was employed on the classification of TNBC and NTNBC samples in the TCGA training and the METABRIC validation data sets. The scatter distribution map revealed that the five-DEG signature could precisely identified TNBC and NTNBC samples both in the TCGA training (AUC = 0.938, 95% CI: 0.897–0.921; Fig 3A and 3C) and the METABRIC validation data sets (AUC = 0.831, 95% CI: 0.749–0.864; Fig 3B and 3D). When compared the predicted classification with the actual group, the overall TNBC predictive percent of this model was 94.08% and 92.91% in the TCGA training and METABRIC validation data sets, respectively (Fig 3E and 3F).

### Screening for independent prognostic clinical models in the TCGA training cohort

**Fig 4:**
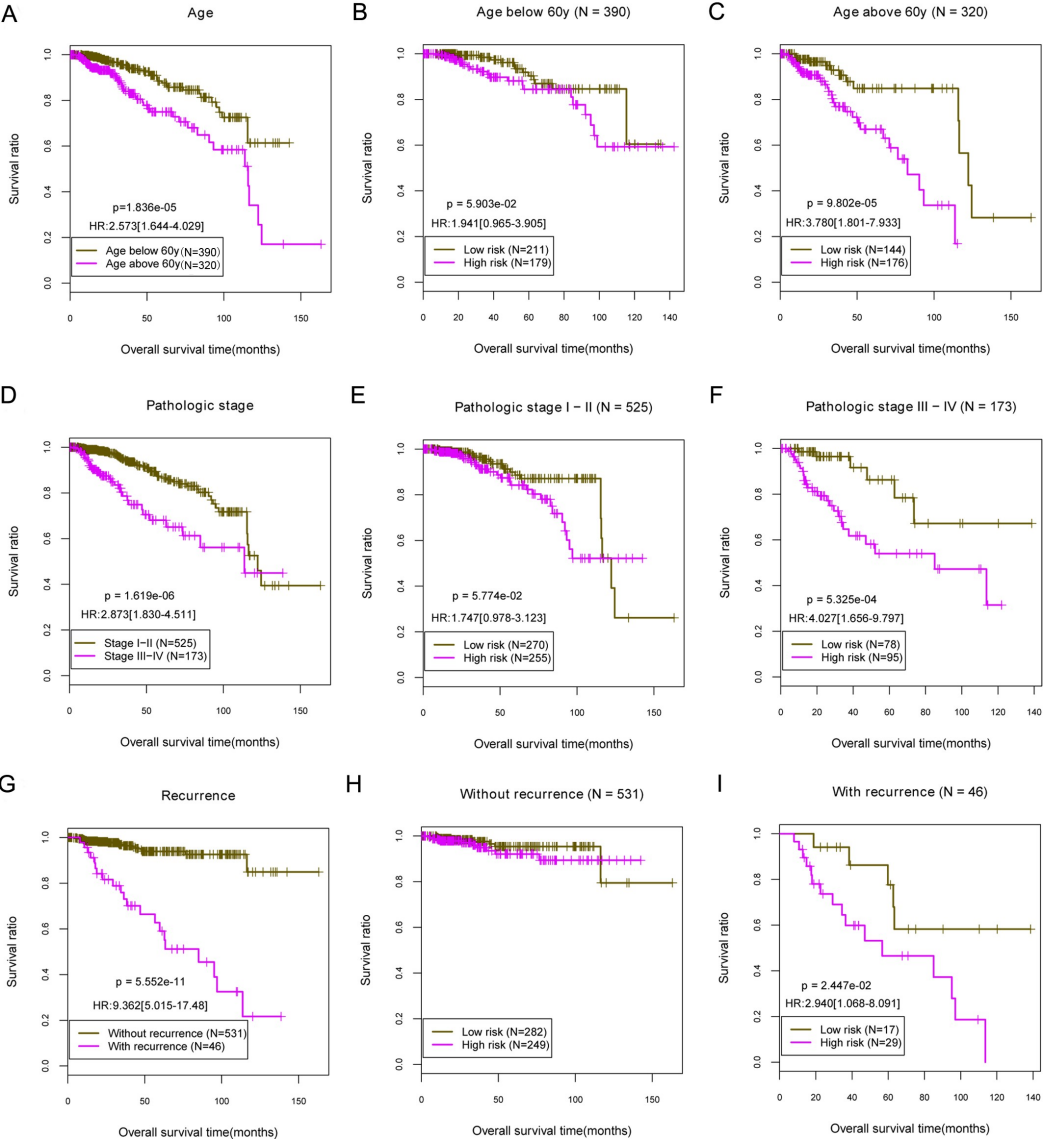
The prognostic-related Kaplan-Meier curve of age, pathologic stage, and tumor recurrence. (A) The prognostic-related Kaplan-Meier curve of age. (B, C) Patients aged over 60 and below 60 prognosis-related Kaplan-Meier curves in TCGA samples. (D) The prognostic-related Kaplan-Meier curve of pathologic stage. (E, F) Pathologic stage I-II and III-IV group in the TCGA sample prognosis-related Kaplan-Meier curve chart. (G) The prognostic-related Kaplan-Meier curve of recurrence. (H, I) Samples of the group with and without recurrence based on the prognostic prediction model Kaplan-Meier curve diagrams. TCGA, The Cancer Genome Atlas.

In the TCGA training set, several clinical independent factors significantly associated with OS were screened out, including the age [HR = 1.060 (1.014–1.109), *P* = 0.00995], pathologic stage [HR = 7.367 (1.168–46.48), *P* = 0.0336], tumor recurrence [HR = 2.237 (1.486–7.129), *p* < 0.0001], and DEG prognostic model status [HR = 2.064 (1.687–6.208), *P* = 0.00197] (Table 3). Then, we stratified samples into subgroups according to age (> 60-year-old and < 60-year-old), pathologic stages (I-II and III-IV), and recurrence (with and without recurrence). Stratified analysis showed that patients aged over 60 [HR = 2.573 (1.644–4.029), *P* < 0.0001; Fig 4A], at higher pathologic stage [III-IV; HR = 2.873 (1.830–4.511), *P* < 0.0001; Fig 4D], and with recurrence [HR = 9.362 (5.015–17.48), *P* < 0.0001; Fig 4G] induced a significantly lower survival rate than those were younger, at lower pathologic stage and without recurrence. OS showed no significant differences between the high- and low-risk groups for patients aged under 60, with early pathologic stage, and without recurrence (*P* > 0.05; Fig 4B, 4E and 4H). As for patients aged over 60 group those who had high-risk scores demonstrated significant lower OS as compared with patients with low-risk scores [HR = 3.780 (1.801–7.933), *P* < 0.0001; Fig 4C]. As for patients in high pathologic stage and recurrence, high-risk scores were correlated with significant lower survival rate than low-risk scores [HR = 4.027 (1.656–9.797), *P* = 0.00053; HR = 2.940 (1.068–8.091), *P* = 0.0224; Fig 4F and 4I].

**Table 3:** Clinical factor screening information table.

Clinical characteristics	Uni-variables Cox			Multi-variables Cox		
	HR	95%CI	*P*	HR	95%CI	*P*
**Age (years, means ± SD)**	1.039	1.023–1.056	<0.0001	1.060	1.014–1.109	0.00995
**Pathologic_M (M0/M1/-)**	7.973	0.829–18.54	0.101	–	–	–
**Pathologic_N (N0/N1/N2/N3/-)**	1.596	1.250–2.038	0.000138	0.439	0.152–1.274	0.130
**Pathologic_T (T1/T2/T3/T4)**	1.550	1.178–2.039	0.00168	0.242	0.081–1.275	0.115
**Pathologic stage (I/II/III/IV/-)**	2.153	1.561–2.971	<0.0001	7.367	1.168–46.48	0.0336
**Radio-therapy (Yes/No/-)**	0.618	0.356–1.071	0.0833	–	–	–
**Target-therapy (Yes/No/-)**	0.246	0.083–0.732	0.0303	1.657	0.180–5.229	0.655
**Recurrence (Yes/No/-)**	9.362	5.015–17.48	<0.0001	2.237	1.486–7.129	<0.0001
**PS model status (High/Low)**	2.509	1.570–4.012	<0.0001	2.064	1.687–6.208	0.00197

Note: CI: confidence interval; HR: hazard ratio; PS, prognostic score; SD, standard deviation; M, metastasis status; N, regional lymph node status; T, tumor status.

### Comparison analysis between prognostic clinical factors and DEGs prognostic risk models

**Fig 5:**
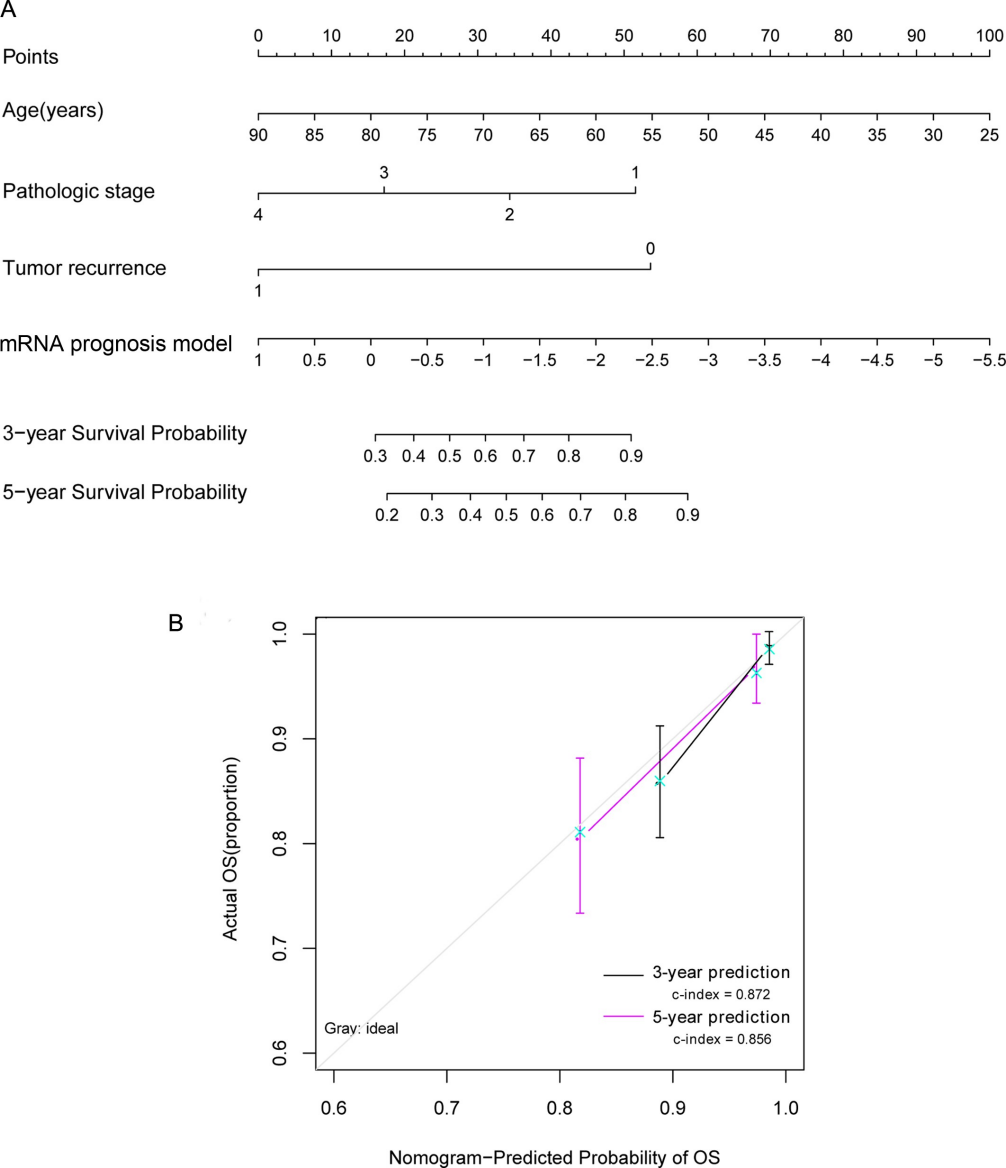
The prognostic nomogram model for independent prognostic factors and three-year and five-year survival prediction. (A) Nomogram of a prognostic model for independent prognostic factors. (B) Line chart of three-year and five-year survival predictions and actual survival. The horizontal axis represents the predicted OS rate, the vertical axis represents the real OS rate, and black and red represent the three-year and five-year forecast line graphs, respectively. OS, overall survival. c-index, concordance index.

The survival nomogram model analysis performed in the TCGA training data set samples revealed that age and DEG prognostic contributed most to the three-year and five-year OS (Fig 5A). The predictive three-year (C-index = 0.872) and five-year (C-index = 0.856) survival probability based on the model was basically in line with the actual survival rates (Fig 5B).

**Fig 6:**
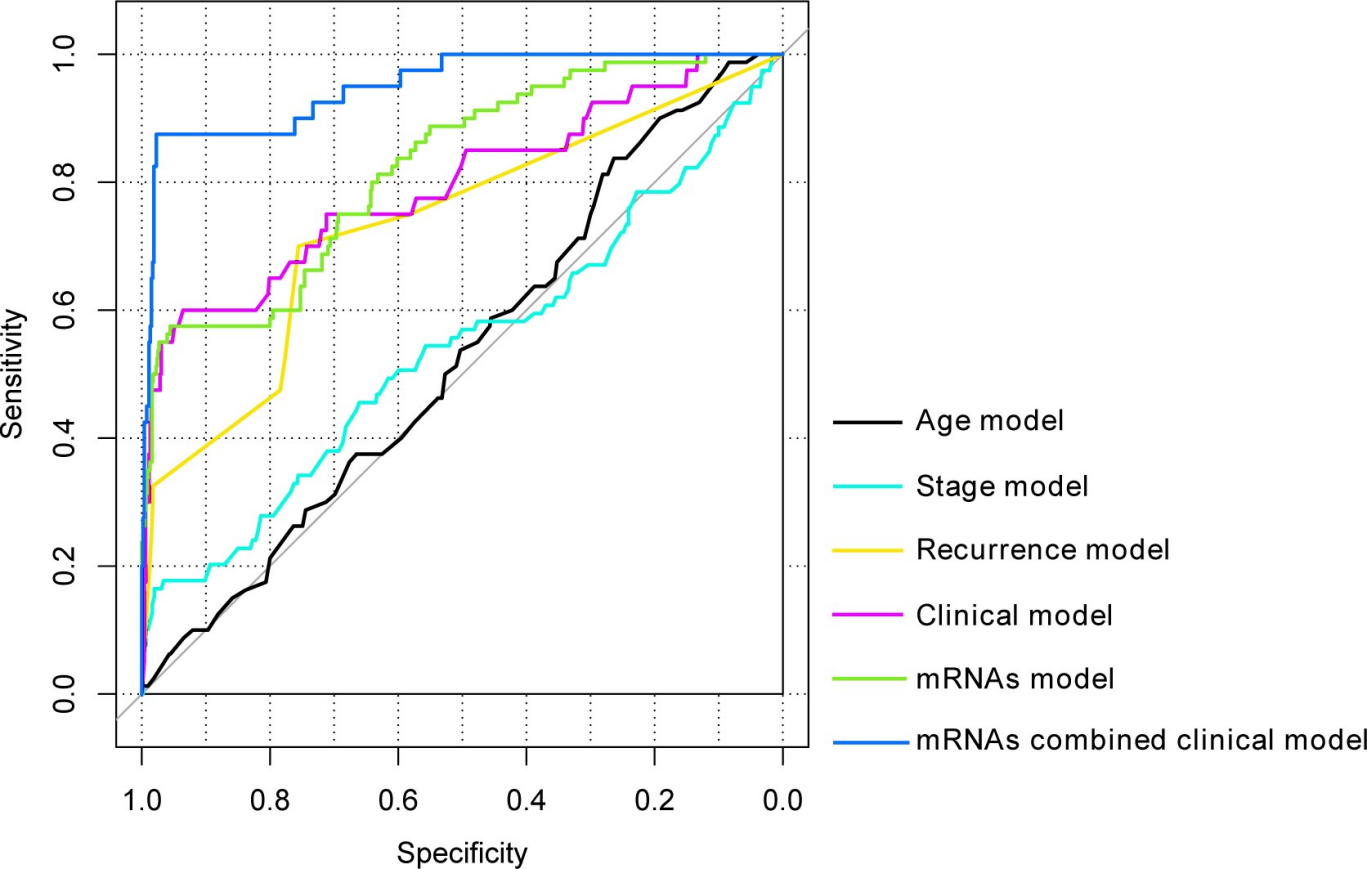
ROC curve comparison based on different models. ROC, receiver operating characteristic. The clinical model contains the factors of age, pathologic, and recurrence.

The model comparison analysis revealed that clinical combination model (AUC = 0.795, *P* < 0.0001; Fig 6; Table 4) presented superior prediction function to single clinical factor ones, including age (AUC = 0.531, *P* < 0.0001), pathologic stage (AUC = 0.540, *P* = 0.00036), and recurrence (AUC = 0.734, *P* < 0.0001). Moreover, the model combined with five-DEGs signature and clinical factors (AUC = 0.950, *P* < 0.00001) exhibited an absolute advantage on predicting prognosis over combined clinical model (AUC = 0.795, *P* < 0.0001) or five-DEG signature model alone (AUC = 0.823, *P* = 0.00039).

**Table 4:** Parameter information of each model.

Models	AUC	C-index	*P*	Specificity	Sensitivity
**Age model**	0.531	0.693	<0.0001	0.563	0.538
**Stage model**	0.540	0.679	0.00036	0.581	0.565
**Recurrence model**	0.734	0.678	<0.0001	0.756	0.700
**Clinical model**	0.795	0.757	<0.0001	0.836	0.701
**mRNAs model**	0.823	0.705	0.00039	0.856	0.757
**mRNAs combined clinical model**	0.950	0.87	<0.0001	0.977	0.875

Note: AUC: the area under the independent ROC curve. C-index: concordence index. The clinical model combined the clinical factors of age, stage, and recurrence.

## Discussion

TNBC is a heterogeneous and aggressive disease with short treatment-to-relapse time and a high rate of visceral metastasis [35]. Its long-term prognosis is worse than other breast cancer subtypes [36]. Worse, TNBC recurrence is known to happen within the first three years [37] after therapy. Once recurrence and metastasis occur, the median survival time is less than one year [38]. Chemotherapy remains the most effective therapy method [13]. In recent years, immunotherapy had been widely studied in cancers, especially TNBC. PD-1/PD-L1 are a pair of immune co-stimulatory molecules contain the medicines of pembrolizumab [14], atezolizumab [15], and durvalumab [16], which were reported to be effective for prolonging OS. Clinical studies have found that immune infiltration could improve prognosis in TNBC patients [17, 18]. Therefore, the identification of DEGs of TNBC may contribute to the in-depth analysis of factors affecting survival. Databases of TCGA, METABRIC, AmiGO 2, and KEGG provide massive comprehensive and reliable high-throughput sequences for mining. In the present study, a five-DEG signature (*CHST4*, *COCH*, *CST9*, *SOX11*, and *TDGF1*) was constructed to predict the prognosis of TNBC. Moreover, the combined prognosis model of DEG signature and clinical factors have a better prediction function than a single DEG signature model or clinical factors.

The signature in our present study consisted of five genes, including *CHST4*, *COCH*, *CST9*, *SOX11*, and *TDGF1*. *CHST4*, enriched in the biological process of immune response, was responsive to any potential internal or invasive threat of an organism [39]. *COCH*, enriched in the biological process of regulating innate immune response positively, is the first line of defense against infection through activating and increasing the frequency, rate, and extent of the innate immune response. *CST9* was enriched in the immune response against microbes mediated through body fluid. *SOX11* was enriched in the biological process of negatively regulation of lymphocyte proliferation by stopping, preventing, and reducing the rate or extent of lymphocyte proliferation. *TDGF1* was enriched in the GO term of cellular response to interferon-gamma. These genes were involved in immune-related GO terms and play essential roles in immune regulation. However, the mechanism of them in TNBC patients still need further analysis.

In recent years, the association between mRNAs, lncRNAs, and the prognosis of TNBC had been widely studied. In the study of Jiang *et al*. [26], one lncRNA-mRNA signature was developed to predict taxane chemotherapy beneficiation and recurrence in TNBC and assist individual frame treatment for TNBC patients. Loibl *et al*. [40] predicted an immune-related mRNA signature TIL and IFN-γ to respond durvalumab in primary TNBC. Ren *et al*. [41] reported a seven-gene signature (1.108*TMEM101–0.213*KRT5–0.315*ACAN–0.464*LCA5+0.446*RPP40–0.373*LAGE3–0.257*CDKL2) on predicting prognostic and de-escalating treatment for early-stage TNBC. In the study of Wang *et al*. [23], they developed a response score with one lncRNA and two mRNAs (2.595*BPESC1–1.09*WDR72–1.428*GADD45A–0.731), which could be employed clinically to predict complete pathological responses in TNBC patients receiving neoadjuvant chemotherapy. Integrated signature of three DE mRNAs (*TRIM59*, *EX01*, and *RAD51AP1*), one DE lncRNA (*KIRREL3-AS1*), and one DE miRNA (hsa-mir-106a) was found to be significantly associated with the prognosis of patients with TNBC [42]. However, the application of these molecular signatures was limited because of the clinical factors and more attention on molecular factors. In this study, the prognostic risk was predicted by a single clinical factor, five-mRNA signature, and combination. The predictive performance of our combined prognostic model was found to be superior to those of the five-mRNA signature, age, stage, recurrence, and clinical alone. Prognostic models should be as simple as possible for patients and doctors to utilize in clinical practice. Moreover, it should be accurate. Our combined prognosis prediction model was based on routine factors, including genetic differences (the five-mRNA signature), baseline demographic factor (age), histopathological characteristic (pathologic stage), and prognostic factor (recurrence). The combined essential factors make the association between risk factors and outcomes more accurate. Consequently, the prognosis risk of TNBC patients can be easily estimated.

A good prognosis prediction model has always been made by considering stratified clinical factors, in which the patients were divided into high and low-risk groups. The prognosis prediction model with stratified factors was more accurate than without. The five-DEG signature performed well on risk stratification in subgroups of pathologic stage III-IV, with recurrence, and those aged over 60. As for the impact of age on prognostic, Liedtke *et al*. [43] demonstrated that patients ≤ 40 years old have poorer survival despite more aggressive systemic therapy, which was 20-years earlier than that demonstrated in our present study. The result might clarify a more accurate signature in our study. As for the pathologic stage, He *et al*. [44] reported that higher stage correlated with longer disease-free survival (HR = 3.13, 95% CI: 1.94–5.06), which was consistent with the result in our study (HR = 2.873, 95% CI: 1.830–4.511). However, our present study was more comprehensive, which consisted of the stage of IV. Moreover, the predictive capacity of factors, including five-DEG signature, age, pathologic stage, and recurrence, were independent of each other.

There are some limitations in the present study. Firstly, we only detected the model but employed in the actual clinical work. Secondly, these genes needed to be further elucidated on the mechanism and functions of TNBC.

## Conclusions

Our present study identified a TNBC prognostic prediction model with five-DEG signature and clinical factors, which will benefit for future research and clinical therapies.

## Supporting information

S1 File The R code of the whole study. (DOCX)

S1 Table The clinical information statistics. The clinical information of overall survival time, age, stage, radiation, targeted-therapy, and recurrence were provided.
(XLSX)

S2 Table The list of differential expressed genes. The thresholds were set as false discovery rate (FDR) < 0.05 and |log_2_ Fold Change| > 1.
(XLSX)

S3 Table The list of differential expressed genes retained for signature identification. (XLSX)

S4 Table The list of differential expressed genes through univariate Cox regression analysis. (XLSX)

S5 Table The list of differential expressed genes involved in the model construction. (XLSX)
