# Supplementary material for: Combined mRNAs and clinical factors model on predicting prognosis in patients with triple-negative breast cancer
Source: PLoS One. 2021 Dec 29;16(12):e0260811. doi: 10.1371/journal.pone.0260811 (PMC8716048; doi:10.1371/journal.pone.0260811)
Supplement: S1 File — (DOCX) [file pone.0260811.s001.docx]

***1. Identification of DEGs***

library(limma)

# load series data from input

gset <- <- read.csv("d:/geneset.txt",header = F,sep = "\t",)

# group names for all samples

sml <- c(rep("NTNBC",594),rep("TNBC",116))

ex <- exprs(gset)

qx <- as.numeric(quantile(ex, c(0., 0.25, 0.5, 0.75, 0.99, 1.0), na.rm=T))

LogC <- (qx[5] > 100) ||

(qx[6]-qx[1] > 50 && qx[2] > 0) ||

(qx[2] > 0 && qx[2] < 1 && qx[4] > 1 && qx[4] < 2)

if (LogC) { ex[which(ex <= 0)] <- NaN

exprs(gset) <- log2(ex) }

# set up the data and proceed with analysis

fl <- as.factor(sml)

gset$description <- fl

design <- model.matrix(~ description + 0, gset)

colnames(design) <- levels(fl)

fit <- lmFit(gset, design)

cont.matrix <- makeContrasts(TNBC-NTNBC, levels=design)

fit2 <- contrasts.fit(fit, cont.matrix)

fit2 <- eBayes(fit2, 0.01)

tT <- topTable(fit2, adjust=“fdr”, sort.by=“B”, number=250)

# load NCBI platform annotation

gpl <- annotation(gset)

platf <- getGEO(gpl, AnnotGPL=TRUE)

ncbifd <- data.frame(attr(dataTable(platf), “table”))

# replace original platform annotation

tT <- tT[setdiff(colnames(tT), setdiff(fvarLabels(gset), “ID”))]

tT <- merge(tT, ncbifd, by=“ID”)

tT <- tT[order(tT$P.Value), ] # restore correct order

tT <- subset(tT, select=c(“ID”,”adj.P.Val”,”P.Value”,”logFC”,”Gene.symbol”))

write.table(tT, file=stdout(), row.names=F, sep=“\t”)

***2. DEGs signature identification and survival prognosis models construction***

A=read.table("d:/geneset.txt",header=T,sep="\t")

sur<-Surv(A[,2],A[,3])

fit=coxph(sur~A[,4],data=A)

.

.

.

.

fit=coxph(sur~A[,167],data=A)

***3. Logit regression model by glm function in R3.4.1***

rm(list=ls())

require(kernlab)

A=read.table("d:/geneset.txt",header=T,sep="\t")

mdl <- glm(formula = Type~.,family = binomial(link = "logit"), data = A)

z <- predict(mdl, A, type = "link")

p <- predict(mdl, A, type = "response")

p <- 1/(1+exp(-z))

pred <- factor(pred, levels= c(0,1), labels = c("TNBC","NTNBC"))

table(A$Type,pred,dnn = c("Labels","prediction"))

modelroc <- roc(pred)

plot(modelroc, print.auc=TRUE, auc.polygon=TRUE, grid=c(0.1, 0.2),grid.col=c("black", "black"), max.auc.polygon=FALSE,auc.polygon.col="white", print.thres=TRUE,lwd=2)

***4. Kaplan–Meier curve method in R3.4.1***

A=read.table("d:/TCGA-PS.txt",header=T,sep="\t")

fit<- survfit(Surv(time,OS) ~status,data = A)

plot(fit, lty = 1:1,col=c("black","red"),xlab="Overall survival time(months)",ylab="Survival ratio",main="Training set")

legend(5,0.2, c("Low risk (N=355)", "High risk (N=355)"), lty = 1:1,col=c("black","red"),lwd=2,cex=0.7)

***5. Screening for independent prognostic clinical factors***

A=read.table("d:/clinical.txt",header=T,sep="\t")

sur<-Surv(A[,2],A[,3])

fit=coxph(sur~age/gender/stage....,data=A)

***6. nomogram analyses***

A=read.table("d:/clinical.txt",header=T,sep="\t")

f2 <- psm(Surv(time,OS) ~clinical factors+status, data = A, dist="lognormal")

dd=datadist(A)

options(datadist="dd")

med <- Quantile(f2)

surv <- Survival(f2)

nom <- nomogram(f2, fun=list(function(x) surv(1095, x),

function(x) surv(1825, x)),

funlabel=c("3-year Survival Probability",

"5-year Survival Probability"))

plot(nom)

cal1 <- calibrate(f2, cmethod='KM', method="boot", u=1825, m=53, B=158)

plot(cal1）
